# Individual differences in auditory scene analysis abilities in music and speech

**DOI:** 10.1038/s41598-025-10263-z

**Published:** 2025-07-05

**Authors:** Robin Hake, Daniel Müllensiefen, Kai Siedenburg

**Affiliations:** 1 Department of Medical Physics and Acoustics, University of Oldenburg, Oldenburg, Germany; 2 Institute for Systematic Musicology, University of Hamburg, Hamburg, Germany; 3 Department of Psychology, Goldsmiths, University of London, London, UK; 4 Signal Processing and Speech Communication Laboratory, Graz University of Technology, Graz, Austria

**Keywords:** Auditory scene analysis, Individual differences, Age, Working memory, Musical training, Hearing loss, Human behaviour, Perception, Auditory system, Cognitive ageing

## Abstract

Auditory scene analysis (ASA) is the ability to organize complex auditory mixtures into meaningful events and streams and is fundamental for auditory perception of both music and speech. Individual differences in ASA are recognized in the literature, yet the factors driving this variability remain poorly understood. This study employs a novel music-based ASA task, the Musical Scene Analysis (MSA) test, alongside a speech-in-noise test, to examine the influence of hearing loss, age, working memory capacity (WMC), and musical training. Ninety-two participants were categorised into four groups: 31 older normal-hearing, 34 older hearing-impaired, 26 younger normal-hearing, and one younger hearing-impaired individual. Results reveal a moderate correlation between ASA performance in speech and music (*r* = − .5), suggesting shared underlying perceptual processes, yet the factors influencing individual differences varied across domains. A dual modelling approach using ridge regression and gradient-boosted decision trees identified hearing loss as the strongest predictor of speech-based ASA, with a weaker effect of age, while musical training and WMC had no impact. In contrast, musical training showed a substantial effect on musical ASA, alongside moderate effects of hearing loss and age, while WMC exhibited only a marginal, non-robust effect. These findings highlight both shared and domain-specific factors influencing ASA abilities in music and speech.

**Supplementary Information:**

The online version contains supplementary material available at 10.1038/s41598-025-10263-z.

## Introduction

Auditory Scene Analysis (ASA) is the fundamental process by which the auditory system organizes complex acoustic environments into meaningful auditory objects and streams, enabling listeners to group, segregate, and track individual sound sources that overlap in time and frequency^1^. In speech, ASA allows individuals to disentangle concurrent sounds, such as separating a single voice from background noise or interfering speakers (commonly referred to as the ‘cocktail party problem’). In the context of multi-source music, this ability - termed Musical Scene Analysis (MSA) - is essential for parsing individual instruments within a mixture (e.g., an oboe within an orchestra) and forms the basis for understanding the rich interplay of instruments, melodies, and harmonies. Individuals are known to vary in their ASA abilities, both in musical and speech dominated acoustic scenes^2–4^; these individual differences - defined as the measurable variability between individuals in perceptual, cognitive, and experiential traits, characteristics, or abilities^5^ - are, however, not yet fully understood. This study aims to identify the key contributors to individual differences in ASA abilities and determine their relative importance.

The literature identifies several potential factors that might contribute to individual differences in ASA abilities. Among these, the influence of hearing impairments (such as the effect of elevated audiometric thresholds) has received considerable attention^6,7^. In fact, the negative effects of hearing impairment on speech perception first tend to become obvious in complex acoustic scenes that place considerable demands on ASA such as having a conversation in a noisy restaurant. Specifically, hearing loss, even in mild forms, has been shown to disrupt the processing of acoustic cues that are essential for separating auditory objects within complex scenes. Such impairments are characterized by compromised frequency resolution, attenuated temporal processing, and diminished spatial hearing capabilities^8–11^. These deficits impair auditory object formation^12^ and the ability to segregate overlapping acoustic streams^13^, resulting in notable difficulties in speech-in-speech and speech-in-noise perception^13,14^. Compared to speech, the role of hearing loss in ASA for music has received less attention. However, hearing-impaired individuals has been shown to experience difficulties in tasks that are associated with successful MSA, such as timbre^4,15,16^, pitch^17,18^, and melody perception^4,19^. Siedenburg et al.^20^ directly examined MSA abilities, demonstrating poorer performance among older hearing-impaired (HI) individuals compared to their younger normal-hearing (NH) counterparts in tracking individual instruments within complex musical excerpts. Survey studies further corroborate these findings, with HI individuals frequently reporting difficulties in discriminating instruments^21,22^. A notable limitation of this research, however, is the confounding of age and hearing loss, underscoring the need for studies that isolate the specific effects of hearing impairment on ASA abilities.

Hearing difficulties and impaired speech perception often persist in the absence of overt hearing loss (i.e., elevated hearing thresholds)^14,23^. This indicates the importance of other factors beyond hearing thresholds in shaping auditory perception, with cognitive processes - particularly working memory capacity (WMC) - emerging as a potential contributor to the observed performance variability among individuals. Working memory, the cognitive system responsible for temporarily storing and manipulating information, allows to hold and process transient auditory details, a capability demonstrably associated with auditory tasks outcomes^24^. Indeed, research has consistently linked WMC to various aspects of auditory perception^25–27^. For example, individuals with better WMC often excel in speech-related tasks^28–30^ and are better able to adapt to difficult listening situations^26,31,32^. Other research linked low-level concurrent sound segregation to attention and WMC^33^. Moreover, WMC is closely associated with listening effort, wherein cognitive resources are allocated to overcome auditory obstacles - a challenge that is significantly greater for individuals with lower WMC^34,35^. Yet, for ASA-related tasks, the link between WMC and performance were not consistent across all listening conditions. For instance, multitalker scenarios versus single speech streams may differentially engage WMC, and factors such as age and hearing status further modulate this relationship^24,36,37^. Furthermore, the extent to which variability in WMC accounts for MSA abilities remains unclear.

Beyond fundamental cognitive abilities, refined listening abilities and knowledge of musical structure, like those acquired by deliberate musical practice may further shape individual perceptual abilities in complex auditory scenarios^38–41^. This reasoning aligns with research demonstrating superior performance of musicians in music-specific tasks like rhythm and beat perception, pitch discrimination, and timbre recognition^4,42–46^. Furthermore, musical training has been linked to a cascade of cognitive benefits, including enhanced information processing speed^47^, enhanced attentional resolution^48^, and even overall improvements in fundamental cognitive regiments like memory^39,49^. This also tends to be associated with enhanced fundamental auditory abilities such as improved frequency discrimination, interaural time differences perception and attentive tracking^50–53^. Musicians also have been shown to outperform non-musicians in auditory stream segregation^54–59^, with some evidence suggesting that musical training may even offer some compensation against age-related decline in auditory sensitivity^60–64^. However, it’s also worth noting that the influence of musical training on speech perception remains under debate^51,65–67^. Furthermore, its specific role in MSA processing also remains a subject of active research: While studies like Siedenburg et al.^4^ and Marozeau et al.^59^ demonstrate an advantage for musically trained individuals in MSA tasks, other investigations failed to establish a link between musical expertise or musical training with MSA performance^68,69^, suggesting a more nuanced relationship or a relatively weak effect that is only observed with sufficient statistical power.

While investigation of peripheral auditory and cognitive factors might offer a unique lens for understanding individual differences in ASA, previous research underscores ageing as a key contributor. Age has been consistently linked to destructive alterations along the auditory pathways and within the cochlea^32,70,71^, with age-related hearing loss emerging as the most prevalent sensory health condition among adults^72,73^. The effect of age extends beyond the auditory periphery, though. Age-related neural degeneration within the central auditory system also affects higher-level auditory processing, compromising timing information, gap detection, sound localisation, and spectral cue processing^26,74–77^ – all of which are essential components for effective ASA^2,78^. These age-related auditory alterations frequently manifest themselves as challenges in filtering out irrelevant competing auditory stimuli^79^ and difficulties with understanding speech in complex listening environments, even when standard audiometric assessments fall within the normal range^6,80^. Alongside these changes, age also brings a decline in cognitive domains crucial for auditory processing. Processing speed, working memory, and selective attention all diminish with age^32,81^, possibly with a negative impact on the ability to detect and track auditory information central to ASA. There is also some evidence that certain age-related auditory effects persist independently of hearing loss and cognitive decline. For example, Lentz et al.^26^ showed that older adults performed worse on several auditory tasks involving temporal processing, even after accounting for hearing loss and working memory. Interestingly, their study also found that stream segregation – a key component of ASA – was the only task that could not be predicted by working memory, hearing loss, or age itself. Certainly, the combined burden of age-related hearing and cognitive changes poses a significant challenge for ASA and warrants careful investigation.

Traditionally, research in auditory perception has primarily concentrated on a limited number of individual factors, often explored in isolation within experiments. However, no single factor can fully explain the variability in ASA performance observed across individuals. Instead, growing evidence highlights the complex interplay between hearing thresholds, cognitive abilities (e.g., working memory), musical training and expertise, and ageing^51,82–86^. To address the complexities inherent in ASA performance, a multifactorial approach is essential - one that examines the relative contributions of these interrelated factors. Such an approach can provide deeper insights into how individuals organise and interpret complex auditory scenes.

Guided by previous literature, the present study investigates several candidate factors to examine their contribution to individual differences in ASA abilities within a single experimental framework: hearing thresholds, working memory capacity (WMC), musical training, and age. ASA abilities were assessed using two domain-specific task. The recently developed Musical Scene Analysis (MSA) test^3^, was used to hear out a target instrument within realistic musical mixtures, while a standardised speech-in-noise task (the Oldenburger Satztest^87^) was employed to evaluate ASA performance in the speech domain, as it requires auditory stream formation and tracking. These tasks were selected for their ecological validity and sensitivity to individual variability in real-world auditory contexts. While the study does not aim to exhaustively capture all potential sources of individual variability, it is based on a diverse sample and a well-defined set of predictors grounded in prior research. We therefore consider the current design well-suited to identifying meaningful individual differences in ASA performance.

We hypothesise that younger individuals, those with better hearing sensitivity (i.e. lower hearing thresholds), stronger WMC, and greater musical training background will demonstrate superior ASA performance. Furthermore, we predict that the factors influencing musical ASA performance will similarly affect speech-in-noise perception, which could be indicative of shared processes for ASA across the music and speech domains.

## Results

**Fig. 1:**
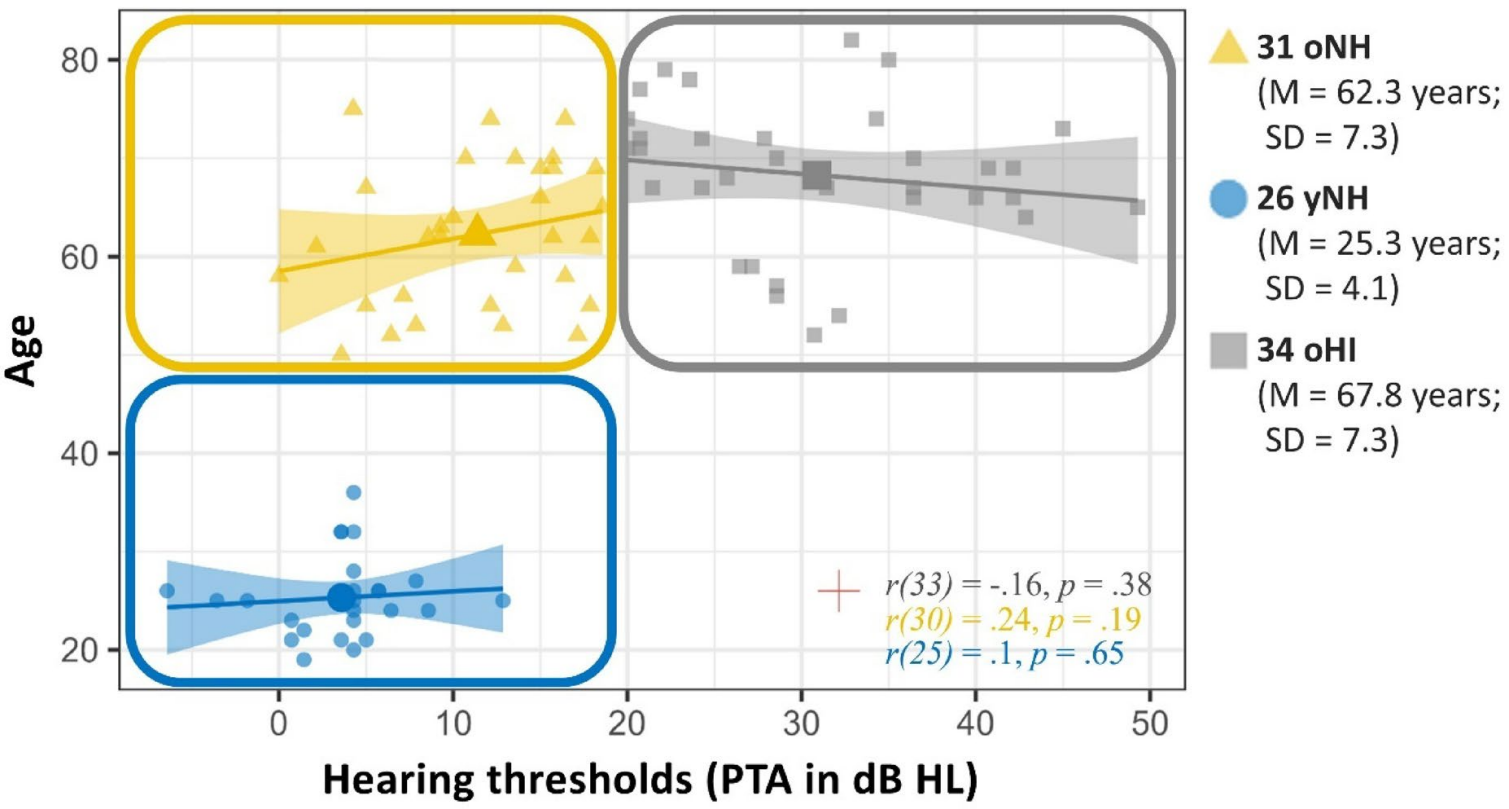
Participant distribution by age and hearing thresholds. Colours indicate thresholds for group categorization (age: 50 years; PTA: 20 dB HL). Scatterplots in each quadrant show group-specific correlations between age and PTA, with regression lines and 95% CIs shaded accordingly. Pearson’s correlation and significance are noted in the lower right corner. Participant groups are as follows: older adults with normal hearing (oNH, yellow, *n* = 31), older adults with hearing impairment (oHI, grey, *n* = 34), younger adults with normal hearing (yNH, blue, *n* = 26). The red cross indicates the single young adult with hearing impairment (yHI, 26 years).

Ninety-two participants were classified into four groups based on age and hearing thresholds (see Fig. 1). Participants between 18 and 38 years were assigned to the ‘younger’ groups and participants with pure-tone average (PTA) thresholds of the better ear exceeding 20 dB HL (averaged across octave-spaced frequencies from.125 to 8 kHz) were classified as ‘hearing-impaired’ (HI). As the yHI group included only one participant, this individual was excluded from all group-based inferential statistics (e.g., ANOVA).

**Fig. 2:**
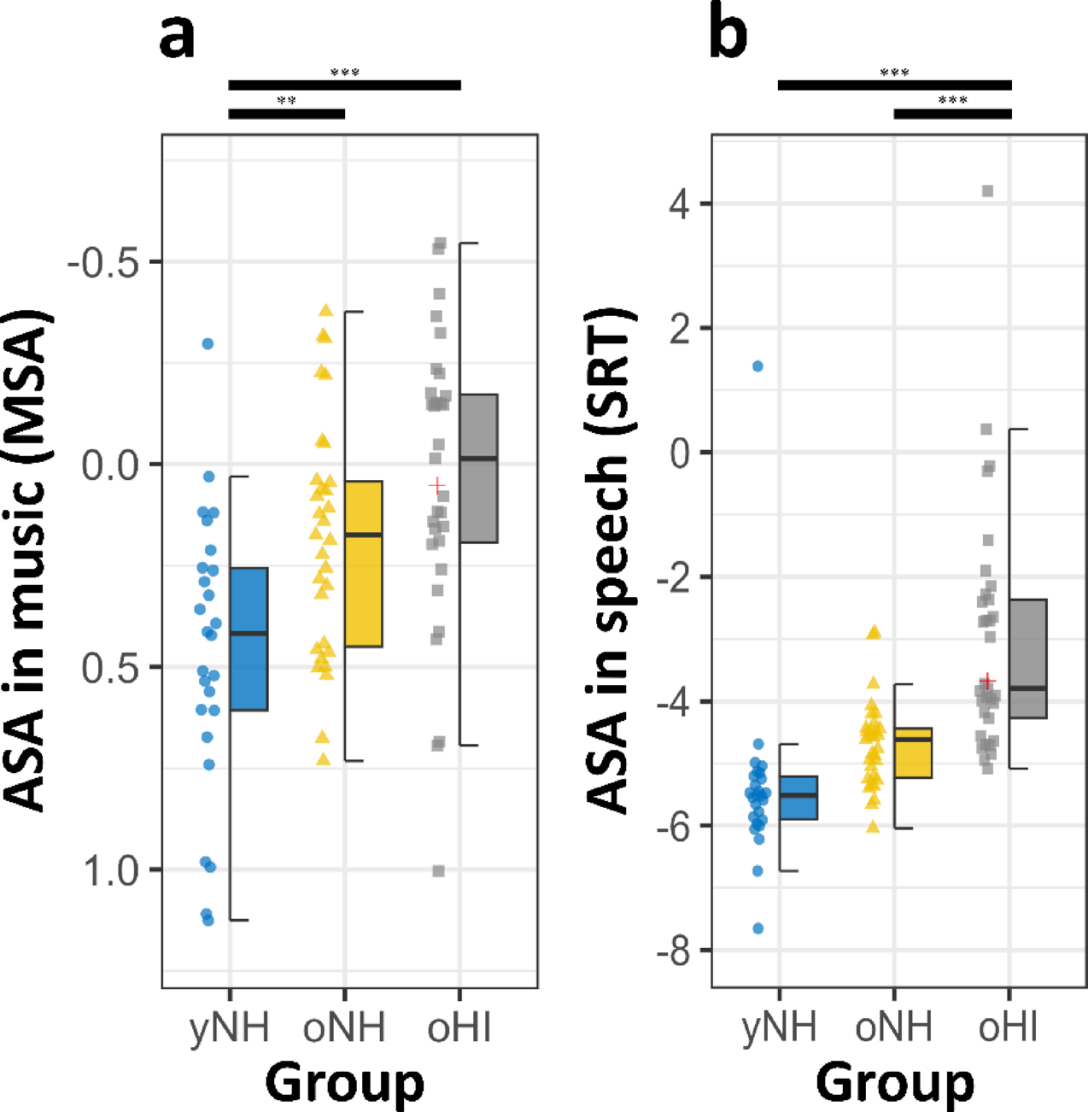
Auditory scene analysis scores for music and speech by groups. (**a**) Musical scene analysis (MSA) scores and (**b**) Speech-reception thresholds (SRT) by groups: Younger normal-hearing (yNH, blue dots), older normal-hearing (oNH, yellow triangle), older hearing-impaired (oHI, grey squares). The red cross marks the single yHI participant. Individual scores are plotted as jittered dots on the left to the boxplots. Higher values indicate better performance for MSA, whereas lower values indicate better performance for SRT. To facilitate visual comparison between domains, the y-axis in (**a**) was inverted, ensuring that better performance is consistently represented by lower values across both panels. Note significance at *p <.05, **p <.01, ***p <.001.

Overall, yNH participants demonstrated significantly better performance compared to both older oNH and OHI individuals in ASA related tasks for both speech and music (see Fig. 2). In the speech domain, yNH participants achieved superior speech reception thresholds (SRT) as measured by the Oldenburg Sentence Test^87^ with a significant effect of group (*F*(2, 85) = 20.13, *p* <.001, *η*² = 0.32). Similarly, in the music domain, performance on the MSA test revealed a significant main group effect (*F*(2, 85) = 11.77, *p* <.001, *η*² = 0.22).

### Modelling individual differences

Despite attempts to match age across the groups of oNH and oHI participants and hearing thresholds across the groups of yNH and oNH during recruiting, small but significant differences remained. Specifically, oHI were significantly older than oNH (*M*_*difference*_ = 5.56 years, *p* =.004). Additionally, yNH demonstrated significantly better hearing thresholds than the oNH group (*p* <.001), with mean pure-tone average thresholds values of the better ear (PTA) of 3.58 (*SD* = 3.79) and 11.39 (*SD* = 5.30), respectively. Furthermore, all examined factors were significantly intercorrelated (see Table 1). These factor intercorrelations complicate isolating the unique contribution of each factor to ASA abilities. For example, the observed strong negative association between PTA and MSA scores (*r* =.49, *p* <.001) might reflect a confound by age-related decline of both hearing and cognition that might influence MSA independently, instead of representing a direct effect of hearing loss.

**Table 1:** The correlation matrix for the test battery.

Variable	MSA	SRT	Age	PTA	Musical training	WMC
MSA	-	− 0.50^***^	− 0.41^***^	− 0.49^***^	0.44^***^	0.35^***^
SRT	84	-	0.47^***^	0.64^***^	− 0.18	− 0.27^*^
Age	88	90	-	0.63^***^	− 0.23^*^	− 0.42^***^
PTA	87	89	90	-	− 0.28^**^	− 0.42^***^
Musical training	82	84	85	84	-	0.37^***^
WMC	82	84	85	84	85	-

Above the diagonal are the Pearson correlations calculated from a complete pair of values, which are significant at *p <.05, **p <.01, ***p <.001. Below the diagonal is the total N for the respective analysis (differences due to missing data). MSA = Musical Scene Analysis; SRT = Speech Reception Thresholds; PTA = Pure Tone Average; WMC = Working Memory Capacity (see Methods for more details).Above the diagonal are the Pearson correlations calculated from a complete pair of values, which are significant at *p <.05, **p <.01, ***p <.001. Below the diagonal is the total N for the respective analysis (differences due to missing data). MSA = Musical Scene Analysis; SRT = Speech Reception Thresholds; PTA = Pure Tone Average; WMC = Working Memory Capacity (see Methods for more details).

To account for intercorrelations among predictors, two complementary statistical approaches were employed: Ridge-penalized linear regression and a gradient-boosted decision tree model (’*XGBoost*’)^88^. By incorporating an L2-norm regularization penalty, ridge regression addresses intercorrelations by shrinking the coefficients of correlated predictors, resulting in more robust and stable model estimates^89^. However, given the plausibility of possible non-linear effects and complex interactions among factors, ‘*XGBoost*’ was employed in addition to the ridge-penalized linear regression model. Importantly, predictor interactions are implicitly considered in ‘*XGBoost*’ models through sequential splits on different factors, allowing the model to capture how the influence of one predictor can vary depending on the value of another.

All participants with complete data, including the single yHI individual, were included in the modelling to support the estimation of independent and interacting effects of age, hearing loss, and other predictors. Both models were used to predict music-related MSA and speech-related SRT scores (see Methods for details on model construction). For MSA, the ridge regression model yielded a root mean square error (RMSE) of 0.30 and an R² of 0.36. The ‘*XGBoost*’ model demonstrated comparable performance, with an RMSE of 0.30 for the training set and an RMSE of 0.31 for the test set, resulting in R² values of 0.34 and 0.31, respectively. For SRT, ridge regression produced an RMSE of 1.4 and an R² of 0.41, while ‘*XGBoost*’ achieved an RMSE of 1.19 (MAE = 0.64) for the training set and 1.21 (MAE = 0.84) for the test set, with corresponding R² values of 0.58 and 0.55. The small differences in RMSE values between the training and test sets indicate that the ‘*XGBoost*’ models generalize appropriate to new data, indicating a relatively low risk of overfitting.

### Hearing loss

**Fig. 3:**
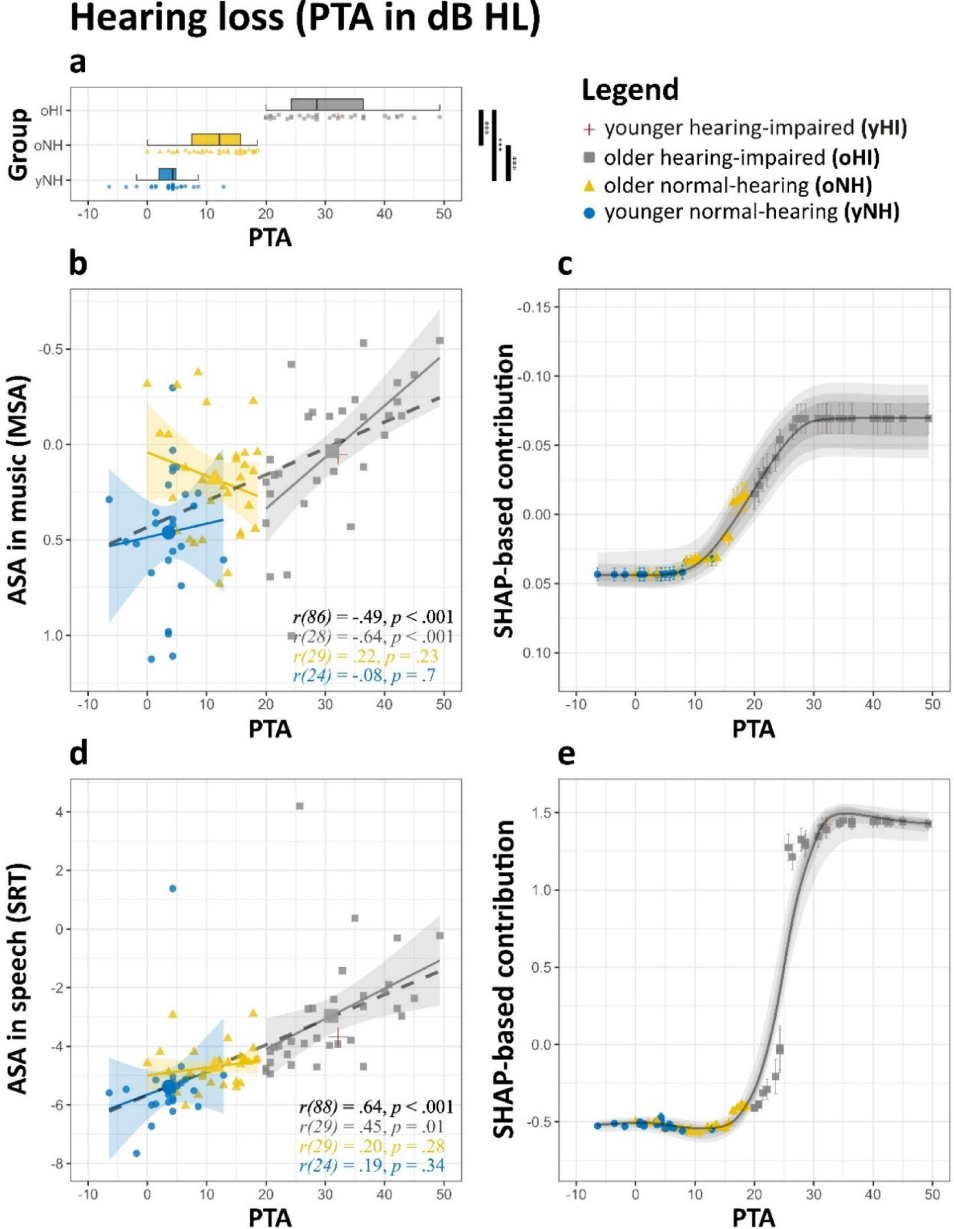
Effect of hearing thresholds on ASA abilities in music and speech. (**a**) Distribution of individual pure-tone average hearing thresholds (PTA) across groups. (**b**, **d**) ASA scores for (**b**) music and (**d**) speech plotted against PTA. Regression lines are fitted for each group, with shaded areas representing 95% CI. **(c**,** e)** SHAP feature contributions for **(c)** music and **(e)** speech plotted against PTA, with positive and negative values indicating their respective impact on model predictions. The LOESS curve (span = 0.35) illustrates the trend, while shaded grey areas show the 95% (light), 80% (medium), and 50% (dark) ranges of LOESS trajectories from the top 1% of models. For interpretability, the y-axis in (**b**) and (**c**) is inverted so that better performance visually aligns across domains. Significance levels: *p <.05, **p <.01, ***p <.001.

In both statistical models, poorer hearing thresholds (higher PTA values) were associated with worse ASA performance in both music (MSA) and speech. Specifically, higher PTA correlated with higher (i.e., worse performance) SRT scores (*r* =.64, *p* <.001, see also Fig. 3) and lower (worse) MSA scores (*r* = −.49, *p* <.001). The ridge regression model identified PTA as the strongest predictor of ASA performance across both domains, yielding standardized coefficients of 0.09 ([0.04, 0.13], *p* <.001) for MSA and 0.96 ([0.68, 1.26], *p* <.001) for SRT. However, while the overall trend was linear, the strength of this relationship varied across groups; that is, PTA had little influence on ASA performance within the normal-hearing range (< 20 dB HL) but became increasingly pronounced as hearing thresholds deteriorated (Fig. 3b, d). The SHAP analysis (SHapley Additive exPlanations) provided by the ‘*XGBoost*’ model corroborated these group-based findings by quantifying the predictor’s marginal contribution to ASA performance. SHAP values reflect the extent to which PTA influences model predictions, independent of other predictors, and are expressed directly in the test score scale (see Methods for details). The SHAP-based trajectory - captured by using a locally estimated scatterplot smoothing (LOESS) regression line - revealed a nonlinear but monotonic trend: PTA had minimal impact below 20 dB HL, followed by a steady increase in its effect up to ~ 40 dB HL, where it plateaued (Fig. 3c, e). For instance, individuals with ~ 40 dB HL exhibited an expected SRT performance 1.6 dB worse than those with a PTA of ~ 23 dB HL, with the latter representing an SHAP score of 0.

### Working memory capacity

**Fig. 4:**
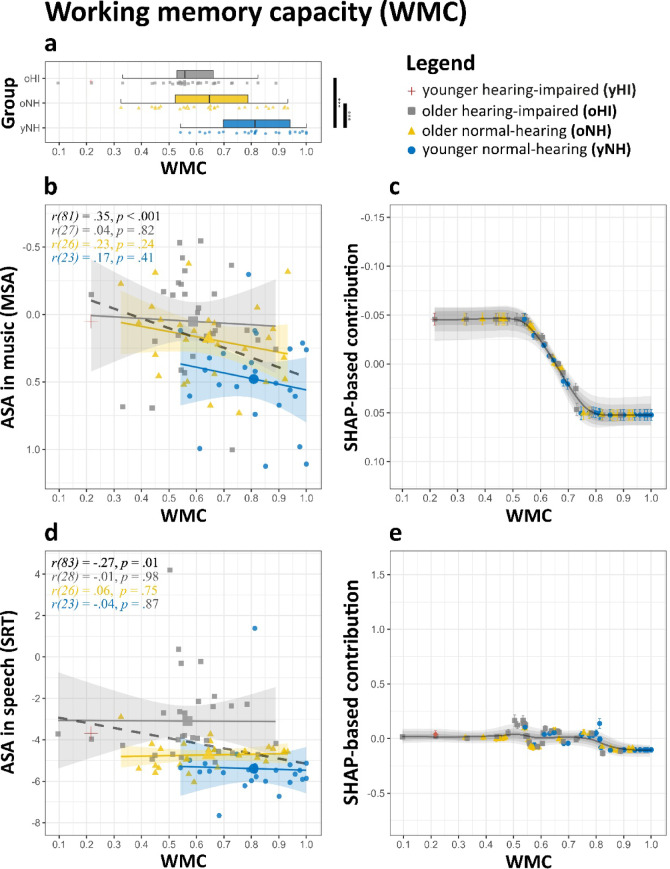
Effect of working memory capacities (WMC) on ASA abilities in music and speech. **(a)** Distribution of WMC across groups. **(b**,** d)** ASA scores for **(b)** music and **(d)** speech plotted against WMC. Regression lines are fitted for each group, with shaded areas representing 95% CI. **(c**,** e)** SHAP feature contributions for **(c)** music and **(e)** speech plotted against WMC. The LOESS curve (span = 0.35) illustrates the trend, while shaded grey areas show the 95% (light), 80% (medium), and 50% (dark) ranges of LOESS trajectories from the top 1% of models. For interpretability, the y-axis in (**b**) and (**c**) is inverted so that better performance visually aligns across domains. Significance levels: *p <.05, **p <.01, ***p <.001.

Working memory capacity (WMC), assessed using the Backwards Digit Span task, correlated significantly with ASA performance in both speech (*r* = −.27, *p* <.05) and music (*r* =.35, *p* <.001, see also Fig. 4). However, these correlations were largely attributable to group membership, defined by age and PTA: Within-group analyses showed no significant correlations between WMC and either SRT or MSA (Fig. 4b, d). The ridge regression model further indicated that WMC did not significantly predict ASA performance in either domain, yielding a coefficient of − 0.03 ([−0.08, 0.01], *p* =.16) for MSA and 0.03 ([−0.18, 0.17], *p* =.90) for SRT. Yet, SHAP-based feature analysis suggested a different interpretation: For SRT, SHAP values indicated no discernible effect of WMC, with a near-flat trajectory suggesting negligible predictive relevance across the observed WMC range (Fig. 4e). In contrast, for MSA, WMC scores revealed a modest but consistent positive effect on performance (Fig. 4c).

### Musical training

**Fig. 5:**
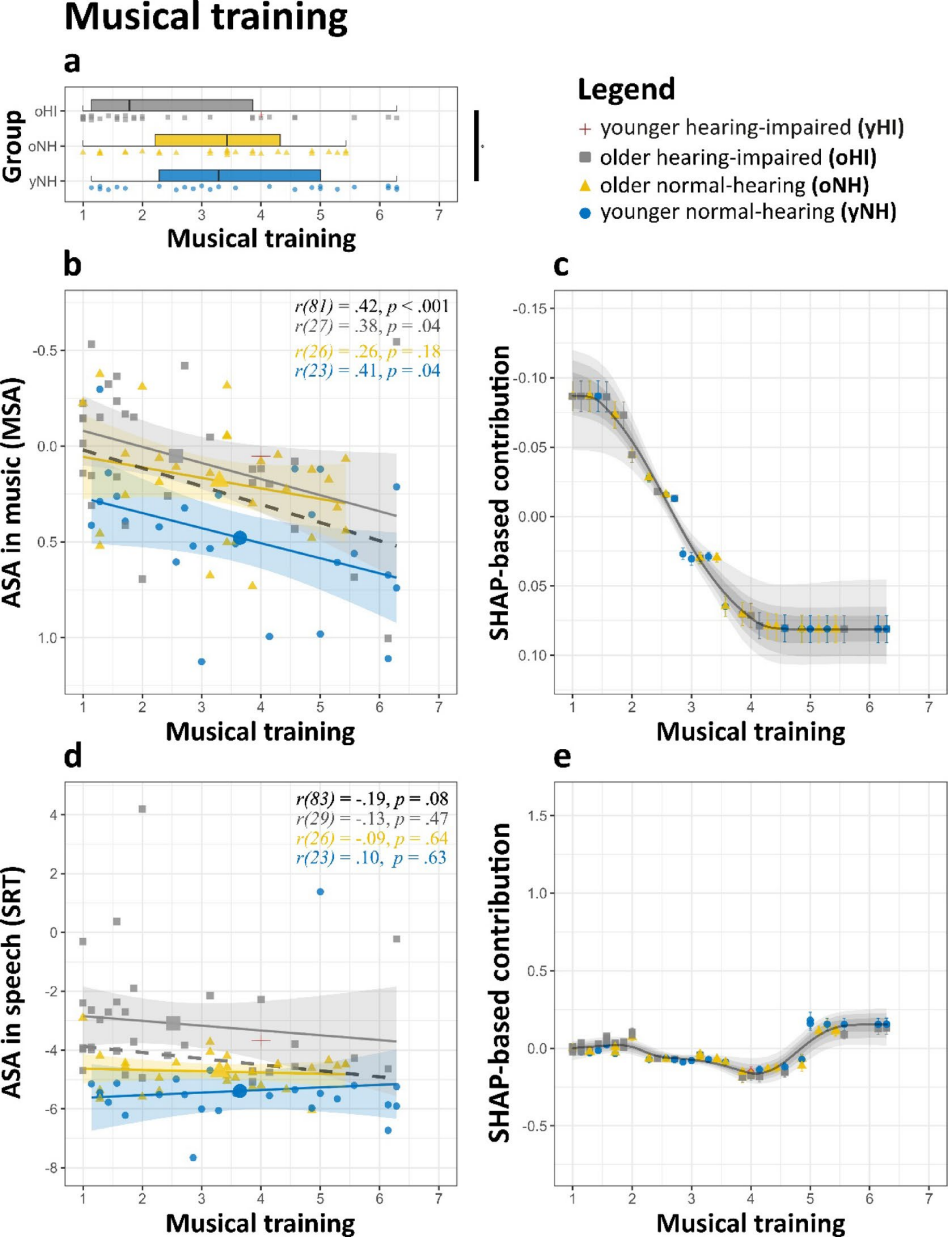
Effect of musical training (Gold-MSI subscale) on ASA abilities in music and speech. **(a)** Distribution of musical training scores across groups. **(b**,** d)** ASA scores for **(b)** music and **(d)** speech plotted against musical training. Regression lines are fitted for each group, with shaded areas representing 95% CI. **(c**,** e)** SHAP feature contributions for **(c)** music and **(d)** speech plotted against musical training. The LOESS curve (span = 0.35) illustrates the trend, while shaded grey areas show the 95% (light), 80% (medium), and 50% (dark) ranges of LOESS trajectories from the top 1% of models. For interpretability, the y-axis in (**b**) and (**c**) is inverted so that better performance visually aligns across domains. Significance levels: *p <.05, **p <.01, ***p <.001.

Musical training, as measured by the Gold-MSI subscale^90^exhibited distinct relationships with ASA performance in speech and music. For speech, no significant correlation was observed (*r* = −.18, *p* =.11, see also Fig. 5). In contrast, for music, a moderate positive correlation emerged (*r* =.44, *p* <.001), with these trends remaining generally consistent within groups (Fig. 5b, d). The ridge regression model further substantiated these findings, indicating that musical training was a significant predictor of MSA performance while showing minimal influence on SRT. For MSA, musical training yielded a standardized coefficient of 0.08 ([0.14, 0.03], *p* =.002), which was comparable in magnitude to PTA. In contrast, for SRT, musical training did not emerge as a significant predictor (*β* = − 0.07 [−0.30, 0.17], *p* =.53). SHAP-based feature analysis further supported these findings. For MSA, SHAP values for musical training exhibited a steady increase across training levels (Fig. 5c). Conversely, in SRT, SHAP values remained near zero across the observed range, suggesting the absence of a meaningful contribution on ASA performance (Fig. 5e).

### Age

**Fig. 6:**
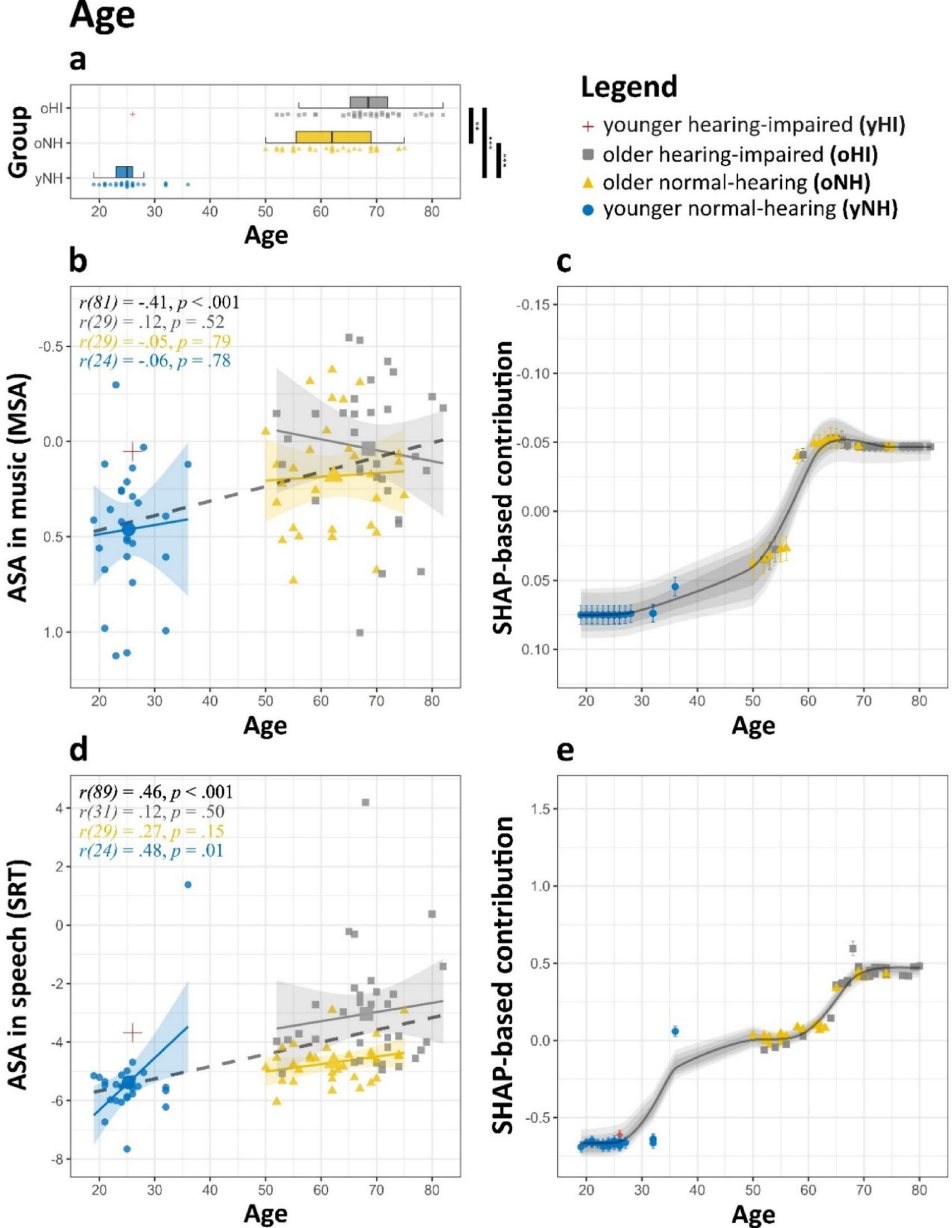
Effect of age on ASA abilities in music and speech. **(a)** Distribution of age across groups (*p <.05, **p <.01, ***p <.001). **(b**,** d)** ASA scores for **(b)** music and **(d)** speech plotted against age. Regression lines are fitted for each group, with shaded areas representing 95% CI. (**c**,** e)** SHAP feature contributions for **(c)** music and **(d)** speech plotted against age. The LOESS curve (span = 0.35) illustrates the trend, while shaded grey areas show the 95% (light), 80% (medium), and 50% (dark) ranges of LOESS trajectories from the top 1% of models. For interpretability, the y-axis in (**b**) and (**c**) is inverted so that better performance visually aligns across domains. Significance levels: *p <.05, **p <.01, ***p <.001.

Age was significantly correlated with ASA performance in both speech (*r* =.47, *p* <.001, see also Fig. 6) and music (*r* = −.41, *p* <.001), suggesting a general decline with increasing age. However, within groups, which are categorised based on PTA and age itself, these correlations disappeared (Fig. 6b, d). The only significant within-group correlation, observed for SRT in the yNH group (*n* = 25; *r* =.48, *p* =.01; Fig. 6b), was driven by a single extreme outlier. Excluding this participant eliminated the effect (*r = −*.03, *p* =.87). Despite the absence of age effects within specific groups, SHAP-based analysis revealed a monotonic increase in its contributions across the full lifespan (Fig. 6c, e) in both domains. Ridge regression results aligned with this pattern, showing a significant effect of age on MSA ( = 0.05, [0.01, 0.10], *p* =.02), while the effect on SRT did not reach significance ( = 0.17, [−0.02, 0.38], *p* =.08). This suggests that, while age effects are less evident within specific subgroups, they manifest across the entire age range.

### Overall factor strength

**Fig. 7:**
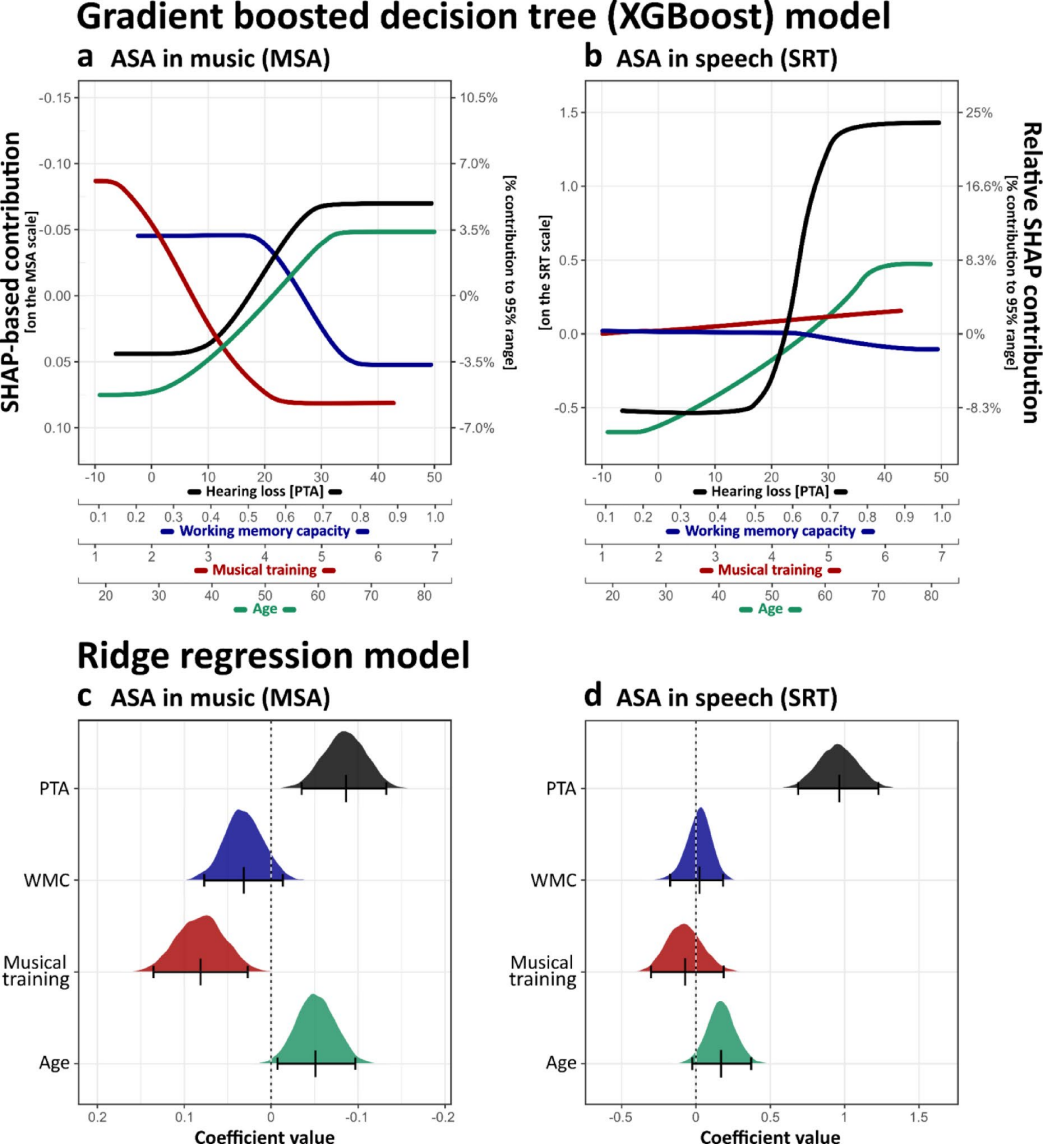
SHAP-based model predictions and ridge regression estimates for ASA abilities in music and speech. **(a**,** b)** SHAP-based feature contributions from the XGBoost model for MSA **(a)** and SRT **(b)**, presented as a simplified (straightened) representation of each predictor’s association with ASA performance. The left axis denotes the SHAP contribution in the original test score scale, while the right axis represents the relative percentage contribution within the central 95% range of observed scores. **(c**,** d)** Ridge regression estimates for MSA **(c)** and SRT **(d)**, showing coefficient distributions, that are established by bootstrap resampling with 1000 iterations. Black markers indicate the 95% CI.

The ‘*XGBoost*’ and ridge regression models largely align in identifying key predictors of ASA performance, though slight differences emerge in the relative magnitudes of associations, particularly for MSA. To quantify these relationships, the SHAP contribution range was evaluated in relation to the listeners performance range on the respective test score scale. Specifically, instead of considering the full observed range of MSA and SRT scores, the top and bottom 2.5% of performers were excluded to remove extreme values (i.e., retain only the central 95% of the distribution). This allowed for an estimate of each predictor’s relative contribution to ASA performance across domains. For instance, a SHAP value of 0.5 for SRT corresponded to approximately 8.3% of the performance range within this adjusted 95% distribution (Fig. 7a, c). Applying this method, musical training accounted for the largest share of variance in MSA (12%), followed by age (10%) and hearing loss (9%), while WMC covered 7%. Ridge regression results showed a similar pattern, ranking hearing loss and musical training as the strongest predictors, with age exerting a slightly weaker effect. WMC showed a small but non-significant association. For speech, both models consistently identify hearing loss as the strongest predictor, with SHAP contributions covering 33% of the observed range. In contrast, neither model detected a meaningful association for WMC or musical training, both of which remained negligible across the observed data range. Age exhibited a weaker association with SRT compared to hearing loss, covering 19% of the SHAP contribution range, but did not reach significance in the ridge model (Fig. 7b, d).

## Discussion

This study investigated a diverse sample of listeners with varying hearing thresholds, WMC, musical training levels, and ages to evaluate the unique contributions of these factors to ASA performance in music and speech. Consistent with prior research, younger NH individuals outperformed all other groups in both ASA tasks, followed by older participants with NH, with older HI individuals performing the worst. Importantly, however, the observed and well-documented interrelationship among the examined factors complicate the interpretation of individual isolated factor contributions^25,85,86^. To address this, two approaches have been employed, one that implies linearity (ridge regression) and another one that does not (called ‘*XGBoost*’). Notably, individual ASA performance in speech and music are moderately correlated (*r* = −.50), suggesting shared underlying mechanisms across both domains (in line with Bregman^1^). Conversely, listener-specific factors exhibited domain-dependent associations with ASA performance, aligning with earlier suggestions of additional and distinct mechanisms for speech and non-speech processing^91^.

### Effect of hearing loss

We identified a strong relationship between hearing loss (PTA in dB HL) and poorer ASA performance in speech, alongside a moderate relationship with MSA. Both the ‘*XGBoost*’ model estimations and group-level analyses indicated that the adverse effects of hearing loss became apparent even in individuals with mild hearing loss (PTA > 20 dB HL), with ridge regression identifying it as the strongest predictor of ASA abilities in both domains. While some research has reported no significant relationship between hearing loss and stream segregation^26^, the present findings align with broader evidence highlighting the critical role of intact auditory pathways for effective ASA across both music and speech^3,10–12^. Discrepancies in prior findings may be a consequence of differences in task design, as many studies have relied on artificial paradigms (e.g., pure-tone ‘ABA’ designs) rather than ecologically valid stimuli. Additionally, the extent to which high-frequency hearing loss is considered may be relevant, as ASA deficits may be more pronounced when thresholds above 4 kHz are taken into account^92^, which are often disregarded in conventional assessments.

Interestingly, MSA appeared more robust to hearing loss compared to speech-based ASA, with the relative impact of hearing loss on speech being approximately three times greater than in music. This difference may be partially attributed to task demands. In the speech task, participants were required to recall exact words embedded in noise. In contrast, the MSA task allowed for the detection of a target instrument based on brief, salient cues. That is, listeners may have benefitted from short temporal gaps in the background mixture—so-called ‘listening in the dips’ opportunities—without needing to track the target continuously throughout the excerpt. If this ability remains at least partially preserved, hearing loss may exert a weaker influence on MSA. However, dip listening in musical scenarios may also be impaired in individuals with hearing loss^4^, which could limit the effectiveness of this strategy.

### Effect of working memory capacities

As listening conditions become more challenging (e.g., increased background noise or increasing the number of instruments in a mixture), auditory processing becomes more effortful, relying increasingly on attentional and WM resources^31,93^. Consequently, we hypothesized that individuals with greater WMC would be better equipped to manage the complexity of musical mixtures, facilitating the retention, rehearsal, and recall of individual instruments. Indeed, while an overall relationship between WMC and ASA performance in both speech and music domains was observed, this relationship was absent within individual groups. Both models further support this, identifying WMC as the least predictive factor of ASA performance overall. Specifically, WMC showed no effect on speech-based ASA. For MSA, model estimates diverged: while ‘*XGBoost*’ suggested a small positive association, the ridge regression model did not yield a statistically significant effect. These results suggest that any potential effect of WMC on ASA is likely minimal, and the current data do not offer strong support for its practical significance.

Nonetheless, this finding is somewhat unexpected, given the well-documented role of WMC in auditory perception^25,27^ and prior evidence linking WMC to stream segregation and speech-in-noise listening performance^94^ particularly among older hearing-impaired listeners^28^. Several factors may explain this discrepancy. One possible explanation is that previously reported associations between WMC and ASA may primarily reflect confounding influences of hearing impairment or aging, rather than a direct causal relationship. For instance, Golden et al.^95^ found no significant differences in ASA abilities in music between Alzheimer’s patients and healthy age-matched controls, despite the former group exhibiting pronounced cognitive impairments, including deficits in WMC^96^. Other evidence suggests the WMC association with ASA to be age-dependent, with no effects being reported among NH individuals^37^ - a pattern consistent with our group-based findings. Alternatively, task-specific factors may account for the weak or inconsistent findings. The speech-in-noise task employed in this study may impose minimal WMC demands compared to other ASA tasks requiring more demanding scenarios, such as multiple talkers embedded in background sounds. Similarly, the weak but visible trend for an effect of WMC on MSA scores may be attributed to the design of the MSA task, which involves a single-item recall paradigm with a short (1-second) delay, thereby relying to a small degree on a working memory mechanism. While the current study assessed working memory in terms of capacity (i.e., the measurable cognitive limit to store units of information), future work should consider a broader conceptualization of working memory, e.g. including attentional control, or broader working memory capabilities, as these factors may better explain variability in ASA performance across speech and music domains. Additionally, future studies could incorporate more demanding ASA paradigms, such as those requiring sustained stream tracking or complex auditory scene recall, to better evaluate the role of WMC in advanced ASA tasks.

### Effect of musical training

Musical practice often involves the identification and discrimination of subtle acoustic details and advance integration of complex auditory streams on a regular basis. Thus, musical training cultivates a unique skill set, potentially offering musicians a distinct advantage within complex auditory scenes like those encountered in ASA tasks^54^. To investigate this, we abstained from a general cut-off score for musicianship, instead considering musical training as a continuum. Our findings revealed a moderate positive relationship between musical training and MSA abilities, independent of hearing loss or age. Specifically, the model suggests that the strength of association of having no musical training compared to extensive musical training on MSA performance is comparable to the impact of mild-to-moderate hearing loss or ageing by approximately 40 years. This finding aligns with research demonstrating the potential of musical training to mitigate age-related auditory decline or declines associated with reduced hearing sensitivity^60–64^. Notably, however, this effect was observed exclusively for musical and not for speech-based ASA. The lack of a clear transfer effect highlights the domain-specific nature of auditory processing, suggesting that skills cultivated through musical education and training may not readily generalise to the unique cognitive and perceptual demands of speech perception in noise. Our findings thus challenge the assumption that musical training universally enhances auditory abilities, raising the question of whether and how its benefits might extend to speech perception - a debate that remains unresolved and polarised in the current literature^51,65–67,97^. However, such associations may depend on task difficulty and listener characteristics, with transfer effects more likely to be of benefit under challenging listening conditions. Follow-up studies should explicitly account for these contextual dependencies^98^.

### Effect of ageing

Age, with its associated changes in hearing sensitivity, neural degeneration, and general cognitive functioning^7^ often serves as a proxy for a multitude of underlying physiological and auditory influences. These factors, however, complicate the isolation of chronological age’s distinct contribution to ASA. To disentangle these effects, we examined a broad age range while controlling for variations in hearing thresholds within groups. At the sample level, we observed a general decline in ASA performance across both speech and music, with increased age and hearing loss. However, within the three groups defined by age range and hearing loss severity, the age effect was largely diminished. Even among older participants (50–80 s), a period typically associated with hearing impairment onset, age was not a strong predictor of ASA performance when hearing loss was accounted for. This suggests that the initial correlations between ASA and age are primarily driven by variability in individuals’ hearing thresholds rather than chronological age itself. Nonetheless, considering the full lifespan, aging manifests a practically relevant negative impact on ASA performance in both MSA and speech-based ASA. However, incorporating additional factors - such as attentional control or processing speed - as well as factors capturing hearing impairments beyond overt hearing loss may further account for the variance in performance that is currently attributed to aging.

## Limitations

While the present study offers valuable insights into individual differences in ASA across music and speech domains, several limitations should be acknowledged. First, while the dataset covers a broad range of ages and hearing profiles, it includes only one younger HI adult. As a result, generalizations to younger HI populations are not feasible on the basis of the present dataset. Second, cognitive screening was limited to the WMC only, however, deficits in attention, processing speed, executive functioning or other undiagnosed cognitive impairments could impact task performance and may obscure true effects by introducing variance that cannot be account for in the model. Given that ageing is often accompanied by cognitive decline, the lack of screening limits our ability to rule out confounding effects of cognitive impairment, particularly among older participants.

In addition, while both ASA tasks tap into the ability to parse acoustic scenes, they likely engage working memory and attentional resources to different extents. Correspondingly, the tasks differ in structure and perceptual demands, limiting the direct comparability of performance across domains. Future research should aim to better align task designs—for example, by evaluating MSA performance alongside multi-talker speech scenarios, or possibly by comparing speech-in-noise perception using the Oldenburger Sentence Test alongside the Music-In-Noise Task^99^—to more clearly dissociate domain-general from domain-specific mechanisms in ASA. It would also be important to incorporate cognitive screening measures and to consider controlling for both the onset and type of hearing loss (e.g., sensorineural, conductive), as well as specific musical perceptual deficits such as congenital amusia.

## Conclusion

This study highlights the complex interplay of factors contributing to individual differences in ASA processes across speech and music domains. While performance in speech and music-related ASA tasks was generally correlated, distinct predictors emerged to be practically impactful for each domain. In speech, hearing loss and age were the most influential factors, whereas in music, musical training played a pivotal role in predicting MSA abilities. Aging and hearing loss had a less pronounced but still notable impact on the performance in the musical ASA task. Working memory capacity showed no effect on speech-based ASA performance and had only a limited influence on the musical ASA task. These findings highlight the critical role of hearing sensitivity in processing auditory scenes across domains, but particularly in speech-related ASA. Conversely, the observed influence of musical training suggests that extensive musical experience may partially mitigate the effects of hearing loss or aging in domain-specific (i.e. music-related) tasks, highlighting its compensatory potential.

## Materials and methods

### Participants

The experiment included a total of 92 participants (47 males and 45 females). Participants were divided into three groups: 31 older adults with NH (M = 62.3, SD = 7.3; 18 female), 34 older adults with HI (M = 67.8, SD = 7.7; 16 female), and 26 younger adults with NH (M = 25.3; SD = 4.1, 11 female). One additional younger adult with HI (yHI; age = 26, male) was excluded from group comparisons but retained in regression-based model analysis. A graphical illustration of individuals’ pure-tone audiometric thresholds can be found in Figure A1 (in Supplementary Materials). If the average pure-tone audiometric measurement for the better ear in a quiet setting surpassed 20 dB HL, subjects were categorised as hearing-impaired (e.g., Humes, 2019).

### Test battery

**Musical Scene Analysis task (MSA)**^3^. The adaptive Musical Scene Analysis test (MSA) is a listening task developed to assess participants’ ASA abilities in the context of realistic musical stimuli. The MSA adopts a ‘yes-no’ paradigm that resembles a 2-alternative-forced-choice task (2-AFC). In each trial, participants hear a two-second excerpt of a single instrument or voice (the target), a one-second silence, and a two-second multi-instrument excerpt (the mixture). Participants are then asked to decide whether the target was part of the mixture or not. The target instrument varies across trials and includes four different instrument types: bass, guitar, lead, and piano. Stimuli are sourced from an open-source music-database (“MedleyDB”), which consists of real-world multitrack music recordings representing a wide range of musical genres (e.g., pop, rock, world/folk, fusion, jazz, rap, classical). All stimuli were presented monaurally. Participant ability is estimated using weighted-likelihood estimation and is projected on a scale of approximately − 3 to + 3. A score of zero corresponds to the median performance of the reference population from the calibration experiment^3^, which predominantly included NH and mild HI individuals. Higher (positive) scores indicate better performance. The study employed two test sets of the adaptive MSA (version 2.4) with 30 items each.

**Backwards Digit Span Memory Test** (**WMC)**^100^). This established working memory assessment requires participants to recall digit sequences in reverse order (e.g., for the sequence 1 2 3 4, the correct response is 4 3 2 1). Digits are presented visually, one at a time. The test comprised sequences of varying lengths (2 trials each of 4-digit and 5-digit sequences; 4 trials each of 6-digit and 7-digit sequences). Performance is measured by the proportion of correctly recalled digits in each sequence. Average results over all sequences are reported.

**Oldenburger Satztest** (**SRT)**^87^. This adaptive speech-in-noise perception test assesses an individual’s ability to comprehend spoken sentences within a background of speech-shaped white noise. Participants have to verbally repeat five-word sentences presented by a male speaker. These sentences were randomly generated from a predefined word class structure (name-verb-numeral-adjective-object), with each word class drawn from a pool of 10 possible word alternatives. The speech level is adaptively varied against a constant noise level to determine the individual’s 50% speech reception threshold (SRT in dB HL). That is, speech level was adaptively adjusted based on word accuracy: decreased by 1.0 dB for five correctly repeated words, 0.5 dB (4 correct), unchanged (3 correct), or increased by 0.5–1.5 dB (2 to 0 correct). The composite score of two test lists of 20 sentences each were used. Lower (negative) scores indicate better performance.

**Pure-tone-average audiometry** (**PTA**). Hearing thresholds were assessed using standard clinical procedures with an Interacoustics AD528 portable audiometer. Pure-tone thresholds were measured at 0.125, 0.25, 0.5, 1, 2, 4, and 8 kHz. Given the established impact of high-frequency hearing loss on complex auditory processing^101,102^ the PTA was calculated as an average across all measured frequencies. Participants with a PTA exceeding 20 dB HL in the better ear were classified as hearing-impaired^103^. A lower PTA indicates better hearing sensitivity.

**Musical training subscale of the Goldsmith Musical Sophistication Index** (**GMSI)**^90^. The GMSI is a self-report questionnaire that assesses several aspects of musical expertise and experience, including a subscale for musical training. Participants responded to questions such as “*At the peak of my interest*,* I practised my primary instrument for _ hours per day*” and rated their agreement with statements like “*I would not consider myself a musician*” (negatively coded). Responses were given either numerically or on a 7-point Likert scale, ranging from 1 (‘Completely Disagree’) to 7 (‘Completely Agree’). The final musical training score is calculated from a 7-item set, with scores ranging from 1 to 7, where higher scores indicate greater levels of musical training.

### Procedure

Ethical approval for the study was obtained from the Ethics Committee at the Carl von Ossietzky University Oldenburg (Drs.EK/2019/092). All participants provided informed consent prior to participation and received compensation at a rate of €10 per hour. All methods used in this study were performed in accordance with relevant guidelines and regulations. Testing was conducted in two phases. The first phase took place in a controlled laboratory setting. Participants were seated in a soundproof booth and stimuli were presented via calibrated equipment consisting of a computer, an RME Babyface sound card, and Sennheiser HD650 headphones. Long-term sound level was set to 75 dB SPL (A), measured with a Norsonic Nor140 sound-level metre using music-shaped noise. Following a demographics questionnaire, participants completed a pure-tone audiometric test and the speech-in-noise assessment. Participants then undertook the first set of a 30-item adaptive MSA test, followed by additional tests, which will not be addressed in this manuscript. The first phase concluded with a second set of the adaptive MSA. The second testing phase was administered online at least 24 h after the initial session among the same participants. This phase included the full GMSI self-report questionnaire and the ‘Backwards Digit Span’ test. Each of the employed tests incorporated a brief training session with practice items. Participants received immediate feedback and could repeat the training as needed. Participants wearing hearing aids were instructed to remove their devices for the duration of the study (N = 19). This decision was made to avoid unwanted acoustic interference when using headphones and to reduce listener-specific variability introduced by hearing aid processing. To ensure audibility, all participants underwent a loudness check not wearing any hearing aids during the training phase of each task. They were explicitly asked whether the sound level was comfortable and intelligible and were invited to adjust the presentation level if needed. Only two participants requested a higher volume (+ 4 and + 5 dB), while five opted to reduce the level. Note that previous research did not find marked effects of level on MSA in these small ranges that all fall within the comfortable listening range of participants^4^. Further details on hearing aid usage history and individual presentation level adjustments are provided in Table A1 (in Supplementary Materials). Overall, the laboratory phase required approximately 80–120 min, while the online phase took 25–40 min.

### Data analyses

All analyses were executed in R (v2023.10.31)^104^. The significance level was set at *p* <.05 for all statistical tests. Pearson’s correlation coefficients were calculated to examine the relationships between SRT and MSA scores and other auditory and cognitive tests, including Oldenburger Satztest, Backwards Digit Span Memory Test, and the subtest for musical training of the Goldsmith Musical Sophistication Index. Analysis of Variance (ANOVA) was conducted to compare the mean MSA and SRT scores across three different participant groups (excluding the one yHI). Post-hoc comparisons were performed using Bonferroni corrections to control for Type I error. Analyses were conducted on available data, with sample size variations explicitly reported. To predict SRT and MSA scores, both ridge regression and gradient boosted decision tree models were employed. Ridge regression applies L2 regularization to mitigate multicollinearity while assuming linear relationships among predictors. In contrast, the decision tree model accounts for both multicollinearity and complex non-linear interactions without assuming linearity. Predictors included the subtest of the GMSI for musical training, age, PTA, and WMC.

#### Ridge regression model construction

Prior to analysis, all predictor variables were standardized, and only complete data were included (MSA: *n* = 82; SRT: *n* = 84). The optimal regularization parameter (L2) lambda parameter was determined via 10-fold cross-validation. The final ridge regression model was then fitted using this optimal lambda (~ 0.11 for SRT and ~ 0.16 for MSA). Bootstrap resampling (1,000 iterations) was applied to estimate the variability of the regression coefficients. Confidence intervals (95%) and two-sided bootstrapped p-values were computed for each coefficient, providing robust inferential statistics. This approach allowed for the assessment of the stability and significance of predictor effects across resampled datasets. The ridge regression model was applied to the full dataset, without holding out a separate test set for validation.

#### Gradient boosted decision tree model (XGBoost)

For the ‘*XGBoost*’ model^88^, the ‘*gbtree*’ booster method was implemented using the ‘*xgboost*’ package in *R* (v1.7.7.1). Complete datasets from participants with available MSA (*n* = 89) and SRT (*n* = 91) scores were included. Model construction employed a random hyperparameter search, an efficient approach for exploring a broad hyperparameter space. Random search is often preferred for hyperparameter optimization as it tests diverse parameter combinations, increasing the likelihood of identifying configurations that minimize validation error^105^. A total of 2,000 random iterations for both MSA and SRT were conducted, with parameter bounds set to balance model complexity and mitigate the risk of overfitting. The specified bounds were as follows: learning rate (*eta*: 0.05–0.15), maximum tree depth (*max_depth*: 3–5), number of boosting rounds (*nrounds*: 50–100), data subsample ratio per boosting round (*subsample*: 0.5–0.9), feature subsample ratio per tree (colsample_bytree: 0.5–0.8), minimum child weight (*min_child_weight*: 1–20), L2 regularisation (*lambda*: 0–1.5), L1 regularisation (*alpha*: 0–1.5), and minimum loss reduction required to make a further split (*gamma*: 0–1.5). The training and test set split was also optimised as part of the tuning process, with bounds set between 0.5 and 0.9. Model performance was evaluated using a combination of Root Mean Squared Error (RMSE) and differences in RMSE and R^2^ between the training and test sets as an indicator of model fit and overfitting risk. To ensure robust RMSE estimation, repeated random sub-sampling validation (also known as Monte Carlo cross-validation) with 500 iterations was implemented, where each iteration involved a different random split of the data into training and test sets. To further ensure hyperparameter suitability, only models with an RMSE and R^2^ difference between the training and test sets below 0.1 were considered. Instead of selecting a single best-performing model, the average of the top 1% (*n* = 20) of models with the lowest RMSE was then used for the analysis. This approach mitigates the influence of hyperparameter variability and enhances the robustness of the final model estimates. This procedure has been done predicting MSA and SRT scores separately.

For MSA, the final hyperparameter estimates were as follows (mean ± 95% CI): learning rate (η = 0.079 [0.069, 0.090]), maximum tree depth (3.7 [3.36, 4.04]), subsample ratio (0.557 [0.542, 0.573]), feature subsample ratio per tree (0.684 [0.660, 0.708]), minimum child weight (16.6 [15.7, 17.5]), L2 regularization (λ = 0.776 [0.629, 0.923]), L1 regularization (α = 0.646 [0.484, 0.807]), and minimum loss reduction (γ = 0.254 [0.175, 0.334]). The average number of boosting rounds was 75.7 [71.0, 80.4], with a data split of 88.3% for training and 11.7% for testing. For SRT, the final hyperparameter estimates were: learning rate (η = 0.082 [0.07, 0.093]), maximum tree depth (4.05 [3.63, 4.47]), subsample ratio (0.746 [0.7, 0.792]), feature subsample ratio per tree (0.693 [0.670, 0.717]), minimum child weight (14.5 [13.1, 15.9]), L2 regularization (λ = 0.926 [0.745, 1.11]), L1 regularization (α = 0.831 [0.615, 1.05]), and minimum loss reduction (γ = 0.803 [0.611, 0.995]). The average number of boosting rounds was 72. [66.1, 78.], with 88.7% of data used for training and 11.3% for testing.

#### SHAP-based model estimates for individual feature contribution

**Fig. 8:**
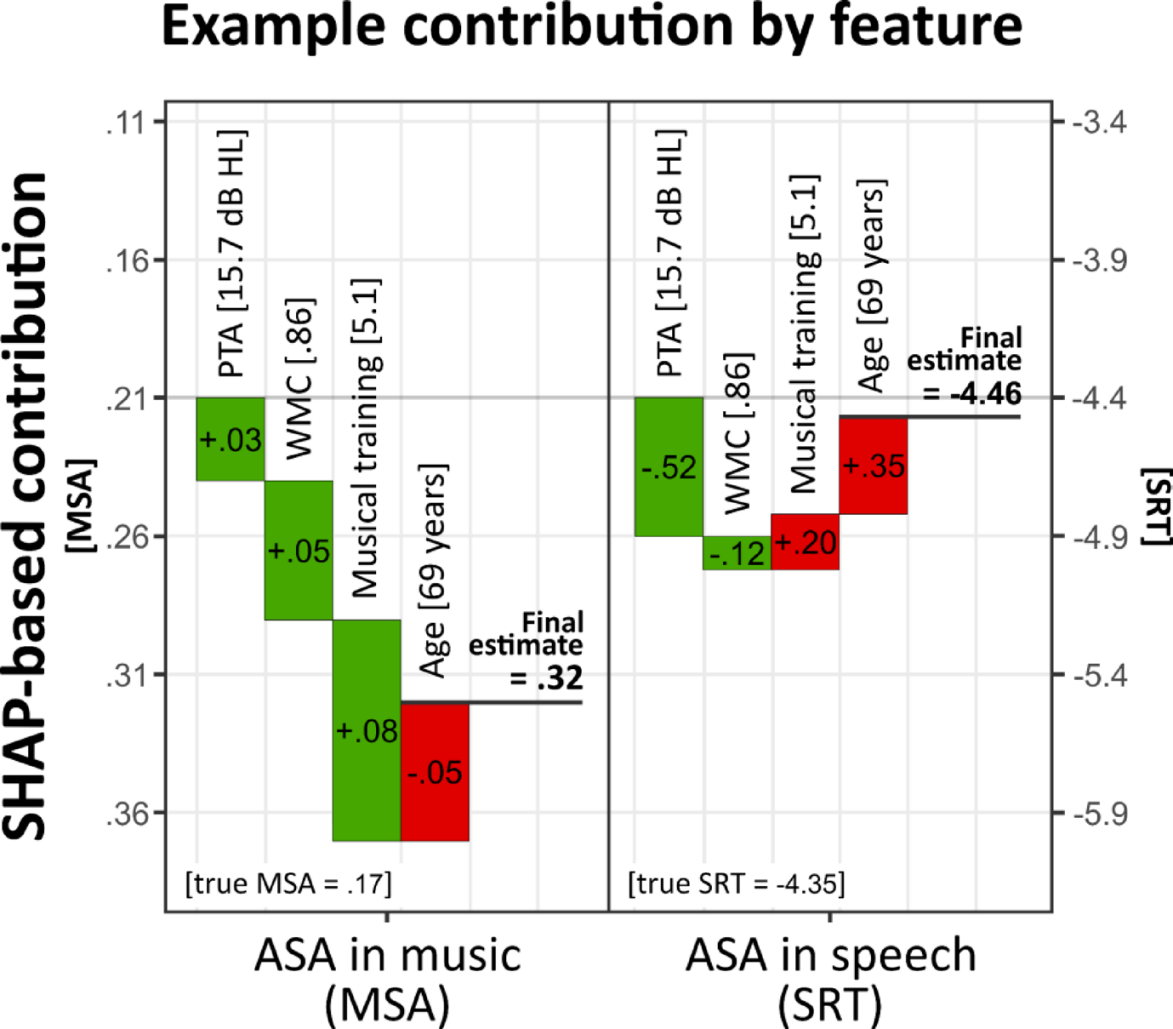
SHAP-based feature contributions for ASA abilities in music and speech. Example SHAP-feature contributions for a single participant (Participant #42) for both SRT and MSA. Positive contributions (green) increase the prediction relative to the baseline value (overall mean), while negative contributions (red) decrease it. Lower scores indicate better performance for SRT, whereas higher scores reflect better performance for MSA.

Importantly, ‘*XGBoost*’ model allows the calculation of SHAP values (SHapley Additive exPlanations), a method for quantifying individual-level feature contributions to model predictions^106^. SHAP values assess each predictor’s marginal contribution by considering all possible combinations of features being present or absent, providing a nuanced interpretation of how individual differences influence model outcomes. Positive SHAP values indicate that a feature increases the predicted score, while negative values suggest a decreasing effect. Since SHAP values are presented on the original test scale, their interpretation follows the scoring direction of each measure. For SRT, lower values indicate better performance, meaning higher SHAP values correspond to poorer outcomes. In contrast, for MSA, higher values denote better performance, so higher SHAP values reflect improved outcomes. The sum of all SHAP values for a participant, combined with the model’s baseline prediction, determines the final predicted score (see Fig. 8 for an illustration). SHAP value estimates and their corresponding LOESS trajectory are constrained to the observed data range and should not be extrapolated beyond it. For instance, PTA values exceeding 50 dB HL fall outside the estimated range and cannot be reliably inferred from the model. Final SHAP estimates were derived from the top 1% of models (*n* = 20) to account for variability in hyperparameter configurations. Confidence intervals (99%) for these estimates were generated through 500 bootstrap iterations, enhancing the robustness of feature contribution interpretations.

## Electronic supplementary material

Below is the link to the electronic supplementary material.

Supplementary Material 1

## Data Availability

The analysis scripts for ridge regression and XGBoost, the sample dataset, and the MSA test used in this study are available in the GitHub repository (https://github.com/rhake14).
