# Supplementary material for: Individual differences in auditory scene analysis abilities in music and speech
Source: Sci Rep. 2025 Jul 5;15:24048. doi: 10.1038/s41598-025-10263-z (PMC12228759; doi:10.1038/s41598-025-10263-z)
Supplement: Supplementary file 1 — Supplementary Material 1 [file 41598_2025_10263_MOESM1_ESM.docx]

# Supplementary Material

## Figure A1


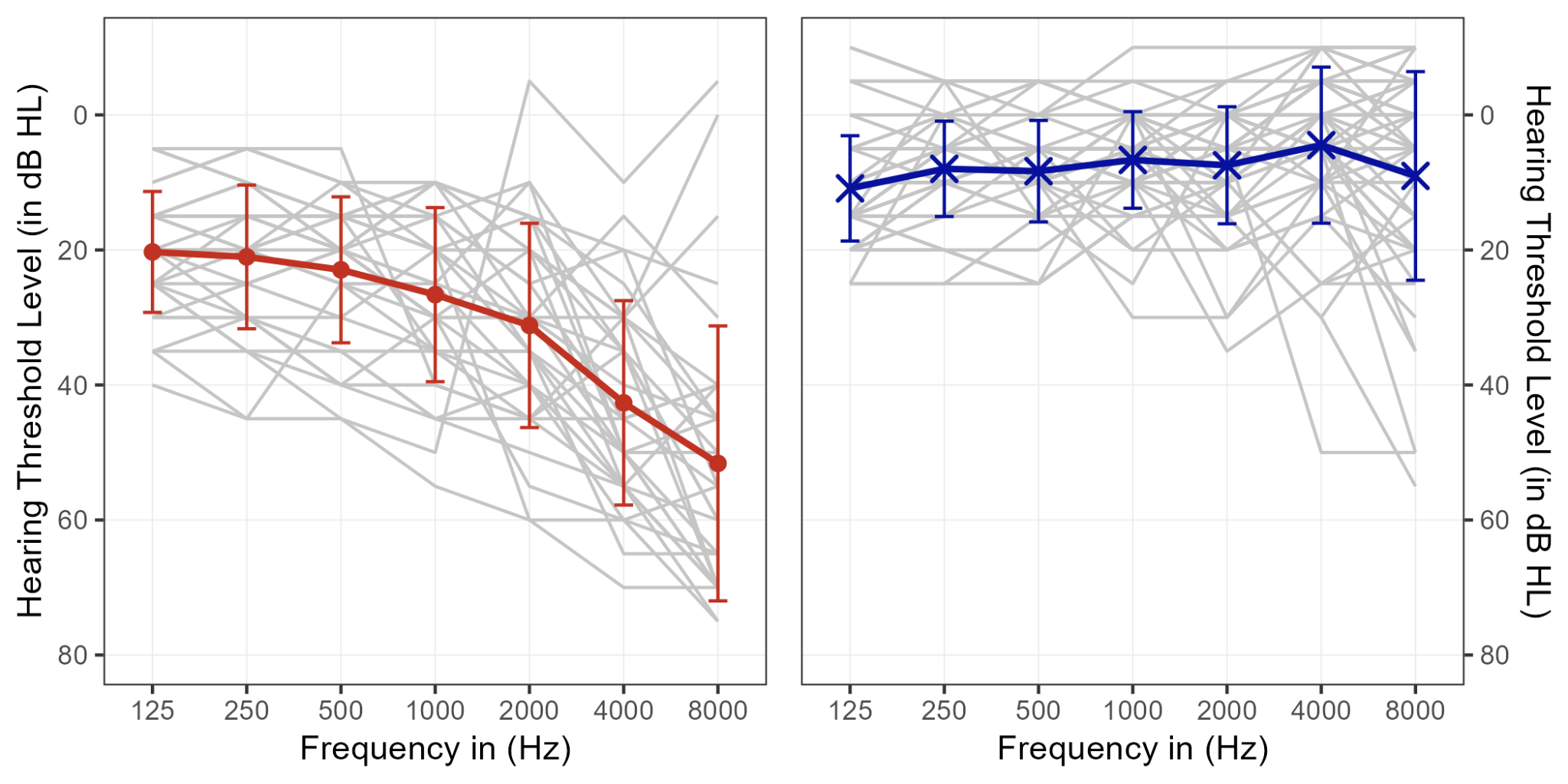
**Figure A1. Pure-tone audiometric thresholds (PTA) for all participants' better listening ear.** Thresholds for individuals with hearing impairment are plotted on the left (red) and for those with normal hearing on the right (blue). A standard clinical ascending-descending procedure in 5 dB HL steps was used. Participants were classified as hearing-impaired if their better ear PTA exceeded 20 dB HL across all frequency bins. The grey lines represent individual participants' thresholds. The bold lines (along SD as error bars) represent the average PTA for each listening group.

**Table A1**
 *Hearing aid use and listening conditions*

|  |  |  |  |  | Hearing aids |  |  |  |
| --- | --- | --- | --- | --- | --- | --- | --- | --- |
| ID | Age | Gender |  | Usage^2^ | Hours^3^ | Years^4^ | Ear noise^5^ | dB adjustment^6^ |
| V2 | 80 | Female |  | Yes | 10 bis 12 | 4 | Yes |  |
| V4 | 58 | Female |  | Yes | 18 | 11 | No | -7 |
| V9 | 81 | Female |  | No | -^1^ | - | No | -7 |
| V10 | 70 | Male |  | Yes | - | 6 | No | +5 |
| V13 | 59 | Male |  | Yes | 10-13 | 4 | Yes |  |
| V15 | 73 | Male |  | Yes | 6 | < 1 | No | +4 |
| V16 | 65 | Male |  | Yes | 14 | 6 | Yes |  |
| V17 | 82 | Male |  | Yes | always | 4 | No |  |
| V22 | 67 | Male |  | Yes | 12 | 6 | Yes |  |
| V30 | 23 | Female |  | No | - | - | No | -5 |
| V33 | 32 | Male |  | No | - | - | No | -6 |
| V41 | 68 | Female |  | Yes | 8 | 6 | Yes |  |
| V73 | 75 | Female |  | Yes | 3 | 4 | No | -7 |
| V76 | 67 | Male |  | Yes | 5 bis 10 | 3 | Yes |  |
| V77 | 66 | Male |  | Yes | 10 | 8 | No |  |
| V80 | 65 | Male |  | Yes | 20 | 9 | Yes |  |
| V82 | 66 | Male |  | Yes | 16 | 12 | Yes |  |
| V83 | 53 | Female |  | Yes | always | 1,5 | No |  |
| V85 | 64 | Male |  | Yes | 14 | 1 | Don’t know |  |
| V86 | 67 | Male |  | Yes | 16 | 4 | No |  |
| V89 | 52 | Male |  | Yes | 17 | 3 | No |  |
| V90 | 69 | Female |  | Yes | 13 | 3 | No |  |

*Note.* ^1^no response were provided; ^2^Do you normally wear hearing aids?; ^3^How many hours a day do you wear your hearing aid(s); ^4^How many years have you been wearing a hearing aid?; ^5^Do you suffer from ringing in the ears / tinnitus / ringing in the ears?; ^6^relative to the standard to 75 dB SPL (A) presentation level. The remaining participants (not listed here) provided no additional comments.
